# Keystone Veterinary Medical Association

**Published:** 1896-07

**Authors:** J. R. Hart, F. S. Bridge, W. L. Rhoads

**Affiliations:** President; Treasurer; Secretary


Secretary.

### KEYSTONE VETERINARY MEDICAL ASSOCIATION.

THE June meeting of the Association was called to order by President John R. Hart, at 8.15, Tuesday evening, the 9th ult., with the following members present: Drs. Thomas B. Rayner, James B. Rayner, Hile P. Eves, Harry J. McClellan, Francis S. Allen, James Johnston, Charles T. Goentner, James T. McAnulty, W. Horace Hoskins, John R. Hart, and W. L. Rhoads.

The Committee on Certificates presented a form for the approval of the members. There were a number of suggestions made, principal among which was that the form so read as to show the date of a member's initiation, also the date of adoption of the certificate. This committee was continued till the September meeting, with instructions to have the certificates at that time.

Dr. Hoskins spoke of a bill before the United States Senate, known as Senate Bill 1552, which prohibited vivisection and experiments with animals. This led to an animated discussion, the sense of the Association being that its passage would cause those who were interested in science to evade or directly violate the law. It was then moved, seconded, and carried that the Association adopt the following resolution, and that the member should transmit copies of same to their representatives through the Secretary:

*Whereas*, believing that the best interests of humanity are served by the judicious permission of experimental research on the lower animals, whereby the value of certain methods of surgical interference can only be determined, and the worth of certain lines of remedies, which at this particular time in the history of medical progress seem to be based upon more exact deductions than ever before, and the wisdom of fully testing these remedies on the lower animals demand no comment from any intelligent, thoughtful person: *Resolved*, that we therefore believe that Senate Bill No. 1552 is calculated to throw around these investigations unnecessary and unjust restrictions, and for these reasons merit our disapproval, and we call upon our representatives from this section to manifest our condemnation of this measure by their voting against the same.

Dr. Allen, as chairman of the lunch committee, now made a report, stating that the verification of what he had said would be found at the restaurant in the Odd Fellows' Temple. It was now moved and carried that the

Association pay for the luncheon, and the Secretary was instructed to draw on the funds for that amount.

A number of communications were now read, Dr. Bachman's being specially interesting and encouraging.

The resignations of Drs. S. J. J. Harger and J. Rein Keeler were now read, voted upon, and immediately accepted by the Association.

The Board of Censors now thoroughly examined the membership roll, and recommended for expulsion the following members for non-payment of dues: R. G. Webster, L. O. Lusson, William B. Werntz, J. T. Ferley, T. Earle Budd, W. T. Edwards, A. F. Schrieber. These expulsions were thoroughly indorsed by the Association.

Dr. Charles T. Goentner cited three cases of stomatitis in dogs, all in the same kennel, about five weeks intervening between the cases. These dogs had been fed on oatmeal and cracklings, and had the best hygienic surroundings. There was a general inflammation of the mucous membranes, and the post mortem showed a profuse catarrhal condition of stomach and upper bowels. Was it contagious?

The report of a case of impaction kindly sent by Dr. E. H. Moore was laid upon the table till the September meeting, as the time was growing late and it was thought advisable to adjourn to the dining-hall, which was immediately done.

Thus part first of the evening's entertainment was finished, and part second began with such alacrity that one might have thought banqueting was the principal aim in life of every veterinarian. Here, too, the zest shown by the participants proved that every true veterinarian (and the membership and friendship of the K. V. M. A. is composed of this class exclusively) believed what was worth doing was worth doing well and immediately. It was also proven during the first course that they were not adherents of the theory that like cures like. Yet from the manner in which the clams disappeared they thoroughly proved that unlike attracts. In fact, throughout everyone proved himself to be an adherent of the old rule, which sayeth, "Eat, drink, and be merry, for to-morrow you may die." And the fact that all escaped gastrotomy was due more to a special act of Providence than any fault of the caterer. This part of the programme was fraught with quite as great an interest as part first had been, and as the evening wore on the participants became even more interested, more enthusiastic, and, strange to say, more eloquent than usual. This (as we are told worldly affairs will) wound up in smoke, and with hearty good-fellowship the meeting adjourned—thus ending one of the most successful years in the history of the oldest local veterinary medical association in continuous existence.

J. R. Hart, President. W. L. Rhoads, Secretary.

F. S. Bridge, Treasurer.

